# New Insights into the Thermal Stability of 1-Butyl-3-methylimidazolium-Based Ionic Liquids

**DOI:** 10.3390/ijms231810966

**Published:** 2022-09-19

**Authors:** Artyom V. Belesov, Natalya V. Shkaeva, Mark S. Popov, Tatyana E. Skrebets, Anna V. Faleva, Nikolay V. Ul’yanovskii, Dmitry S. Kosyakov

**Affiliations:** Core Facility Center “Arktika”, Northern (Arctic) Federal University, nab. Severnoy Dviny, 17, 163002 Arkhangelsk, Russia

**Keywords:** ionic liquids, 1-butyl-3-methylimidazolium, thermal stability, degradation products

## Abstract

One of the most promising applications of ionic liquids (ILs) with 1-butyl-3-methylimidazolium (bmim) cation is based on their unique ability to dissolve and fractionate lignocellulosic biomass, allowing for the development of green biorefining technologies. A complete dissolution of lignocellulose requires prolonged treatment at elevated temperatures, which can cause the partial degradation of ILs. In the present study, a combination of various analytical techniques (GC-MS, HPLC-HRMS, 2D-NMR, synchronous thermal analysis) was used for the comprehensive characterization of bmim acetate, chloride, and methyl sulfate degradation products formed at 150 °C during 6- and 24-h thermal treatment. A number of volatile and non-volatile products, including monomeric and dimeric alkyl substituted imidazoles, alcohols, alkyl amines, methyl and butyl acetates, and N-alkylamides, was identified. By thermal lability, ILs can be arranged in the following sequence, coinciding with the decrease in basicity of the anion: [bmim]OAc > [bmim]Cl > [bmim]MeSO_4_. The accumulation of thermal degradation products in ILs, in turn, affects their physico-chemical properties and thermal stability, and leads to a decrease in the decomposition temperature, a change in the shape of the thermogravimetric curves, and the formation of carbon residue during pyrolysis.

## 1. Introduction

Room temperature ionic liquids (ILs), having melting points below the conventional limit of 100 °C, are considered “green” solvents and reaction media due to their extremely low vapor pressure, non-flammability, low toxicity, and the possibility of regeneration in technological processes [1,2]. Specific physical and chemical properties associated with the ionic nature of ILs, along with the high reactivity of cation and anion devoid of molecular solvation shells, ensure an exceptionally high dissolving power of ILs towards various substances and, first, biopolymers [3]. One of the most exciting applications of ILs is based on their unique ability to dissolve (partially or even completely) lignocellulosic biomass, allowing for its fractionation into polysaccharide and lignin components. Such processes form the basis of the promising environmentally friendly technologies for processing renewable plant biomass (biorefining) as a source of various chemicals and functional materials, as an alternative to fossil hydrocarbons. In this regard, ILs based on the combination of 1-butyl-3-methylimidazolium (bmim) or 1-ethyl-3-methylimidazolium (emim) cations with chloride, methyl sulfate, and acetate anions have found the greatest use [4,5,6,7] due to the suitable melting points and availability on a semi-industrial scale with quite a simple synthesis procedure from imidazole.

The complete dissolution of lignocellulosic biomass in these ILs requires prolonged (up to 12–24 h) treatment at elevated temperatures (100–170 °C) [8,9]. The dissolution rate increases with temperature, the upper limit of which is limited by ILs’ thermal stability. According to the thermogravimetric analysis (TGA) in dynamic mode, the region of active thermal decomposition of bmim (emim) chloride, methyl sulfate, and acetate lies above 200 °C [10,11,12]. However, slow degradation processes causing significant (up to 15%) weight loss after several hours of being kept under isothermal conditions were observed even at significantly lower temperatures (120–150 °C) [10,13,14]. The constant flow of inert gas in the TGA furnace during analysis prevented the accumulation of volatile and semi-volatile degradation products in the IL sample, and, thus, facilitated the weight loss. On the contrary, the use of bulk volumes of ILs in plant biomass fractionation procedures without purge gas results in the accumulation of all formed thermal degradation products in the liquid phase, although their emission into the gas phase was also observed [15]. Among them, aliphatic alcohols, alkyl imidazoles, and aromatic and aliphatic amines derived from IL’s cation predominated. Anion plays a key role in the IL degradation, including through interactions with cation or its decomposition products. They proceed primarily with the elimination of alkyl groups in the form of corresponding alkyl halogenides or alkyl acetates (for halogenide and acetate anions, respectively) and alkyl imidazole formation via the S_N_2 mechanism [16,17]. Another reaction pathway is associated with the deprotonation of bmim or emim, resulting in the formation of thermally labile and reactive imidazolium-based N-heterocyclic carbenes (NHCs) [18,19]. Obviously, this process strongly depends on the basicity of the anion, which decreases in the series: acetate > chloride > methyl sulfate [20,21,22]. Thus, [bmim]OAc demonstrates much lower thermal stability compared to [bmim]Cl and [bmim]MeSO_4_, since chloride and methyl sulfate anions are not basic enough to form significant equilibrium concentrations of NHC in IL [23,24].

A complete list of dialkylimidazolium ILs decomposition products has not yet been established. This is especially true for compounds formed and accumulated in IL as a result of prolonged heating at temperatures below the point of IL active decomposition, which are most important from the practical point of view. The accumulation of degradation products, which may include toxic and corrosive substances, can negatively affect the equipment, plant biomass dissolution kinetics, properties of the obtained products, reusability of ILs, and environmental friendliness of IL-based technology. Moreover, highly reactive IL decomposition products can serve as active reagents covalently binding to plant biopolymers. This may explain the presence of significant amounts of nitrogen in lignins obtained by wood dissolution and fractionation in ILs [7,25]. Another important aspect of this problem is associated with some decomposition products (such as amines [15]), capable of acting as nitrogenous bases contributing to the formation of NHC, thus reducing the thermal stability of ILs.

In the present work, a combination of analytical techniques, i.e., headspace and pyrolytic gas chromatography–mass spectrometry (HS-GC-MS and Py-GC-MS), high-performance liquid chromatography–high-resolution mass spectrometry (HPLC-HRMS), two-dimensional (2D) NMR spectroscopy, and synchronous thermal analysis (STA) were used for the most complete characterization of the thermal decomposition products of bmim-based ILs with a high potential for use in lignocellulosic biomass processing. It is aimed at studying the effect of preheating at 150 °C, as a typical temperature for biomass processing, on the thermal stability and chemical composition of bmim acetate, methyl sulfate, and chloride, revealing the role of IL’s anion in thermal degradation processes.

## 2. Results and Discussion

### 2.1. Volatile and Semi-Volatile Products of ILs Thermal Degradation

**Figure 1:**
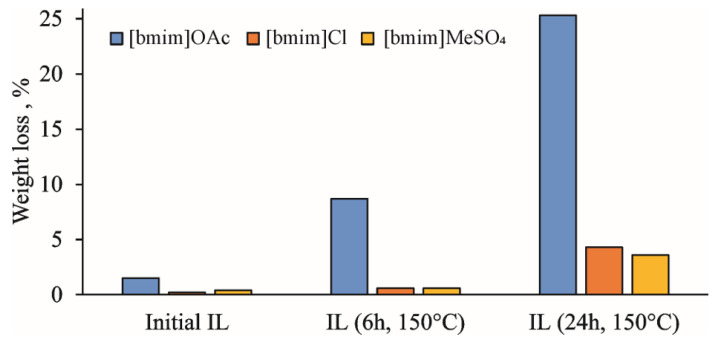
Weight loss of IL samples during vacuum drying at 60 °C.

The active accumulation of volatile degradation products during the thermal treatment of IL at 150 °C is clearly illustrated by the weight loss upon holding the samples in a vacuum oven at 60 °C overnight (Figure 1). In the case of [bmim]OAc, it reaches 8 and 25% for treatment duration of 6 and 24 h, respectively. The amounts of volatile products were different for the studied ILs, which can be arranged according to the weight loss in the following sequence: [bmim]OAc >> [bmim]Cl > [bmim]MeSO_4_. This is in complete agreement with the aforementioned concept of the anion basicity effect on ILs’ thermal degradation due to the formation of the NHC intermediate [17,18,19].

GC-MS techniques allow for drawing conclusions about the composition of the major volatile degradation products of the studied ILs and changes in its relative content after thermal treatment. For GC-MS analysis of the formed compounds, two approaches were used. The first one was based on the study of equilibrium vapor phase (headspace) above ILs subjected to thermal treatment at 150 °C. The obtained results (Table 1) demonstrate the formation and accumulation in ILs of twelve volatile and semi-volatile compounds.

The degradation of [bmim]OAc leads to the formation of ten major products detected in the headspace. Among them, methanol and methyl acetate predominate, indicating the course of bmim dealkylation reactions, including those involving residual amounts of water in the IL. The possibility of *n*-butyl group elimination, alternative to demethylation, is confirmed by the formation of *n*-butanol and butyl acetate, the chromatographic peaks of which are distinguished by much lower intensity due to lower volatility of these compounds compared to methylated analogues. Accordingly, during thermal treatment, the contents of methyl- and butylimidazole in IL increase rapidly. Another, albeit less probable, pathway for the bmim NHC decomposition involves the imidazole ring cleavage, resulting in the formation of trialkylamines (trimethylamine and N,N-dimethylbutylamine). It is worth noting that, along with alkylamines, N-alkylamides were also formed. Two representatives of this class were found in the headspace—N-methylacetamide and N-methylformamide. The reason for their formation can be side oxidative processes, as well as secondary reactions involving the acetate anion.

The range of the detected [bmim]Cl volatile transformation products comprises five compounds. In addition to the already mentioned methanol and 1-alkylimidazoles, 1-chloromethane and 1-chlorobutane were found in the headspace in significant amounts. Moreover, 1-chloromethane gives the peak with the highest signal intensity on the chromatogram, which, however, is due to the high volatility of this compound. The formation of 1-chloroalkanes, as well as alkyl acetates in the case of [bmim]OAc, is obviously associated with the known reaction of IL anion with alkyl moieties in the bmim cation, leading to the formation of 1-alkyl imidazoles, and proceeding through the nucleophilic substitution S_N_2 mechanism [11]. A similar mechanism can be involved in the interaction of the bmim with residual water, leading to the formation of methanol and butanol.

As was expected, [bmim]MeSO_4_ demonstrated the highest stability—of all the nitrogen-containing products, only trace amounts of 1-methyl-1-H-imidazole were found in the headspace. Surprisingly, the second (and last) detected compound, methanol, was observed at a level close to that for [bmim]OAc. This can be explained by its formation not as a result of the bmim cation degradation, but due to the demethylation of the methylsulfate anion in the presence of residual amounts of water.

To evaluate the effect of thermal pretreatment on the course of the ILs’ thermal degradation, additional experiments were carried out using the Py-GC-MS technique. The chosen pyrolysis temperatures corresponded to the maximum rates of ILs’ decomposition (according to STA experiments), and amounted to 242, 290, and 363 °C for bmim acetate, chloride, and methyl sulfate, respectively. The chemical composition of the resulting pyrolysis products (Table 2) is generally similar to that obtained by headspace analysis, and differs in the presence of a few compounds. N-butyl-amides and sulfur dioxide were found in small amounts among the pyrolysis products of bmim acetate and methyl sulfate, respectively, whereas trialkylamines were not detected at all. Another peculiarity of [bmim]MeSO_4_ is the formation of 1-methoxybutane, which has already been observed earlier [15] and can be a result of *n*-butanol methylation with methyl sulfate anion. It is notable that there was a significant increase in the amounts of most detected pyrolysis products and, as a consequence, the total intensity of the peaks in the chromatograms, with an increase in the duration of IL thermal treatment. This can be explained by the rapid evaporation of the volatile products accumulated in the IL when the sample is heated to the target pyrolysis temperature without decomposition to the simple gaseous compounds (nitrogen, ammonia, methane, etc.) that are not retained in the cryo-trap and chromatographic column. Another factor is the change in the pyrolysis conditions due to the presence of degradation products; for example, highly basic amines, which can promote NHC formation.

**Figure 2:**
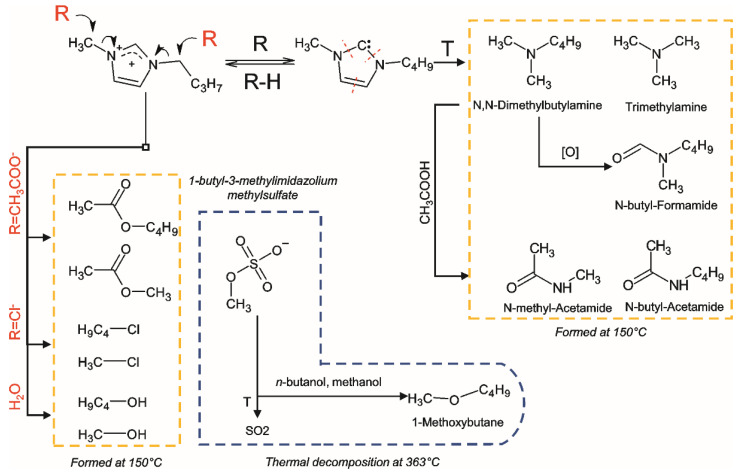
Thermal degradation pathways of the bmim-based ILs with the formation of volatile and semi-volatile compounds.

Summarizing the above, the primary reactions occurring during thermal degradation of the studied ILs involve the interactions with anion by the S_N_2 mechanism, as well as the formation and further decomposition of NHC according to the scheme presented in the Figure 2.

### 2.2. Less Volatile Products of ILs Thermal Degradation

The application of two-dimensional NMR spectroscopy (Supplementary Figures S1 and S2) in combination with the ACD/Labs expert software system for the analysis of [bmim]OAc subjected to 24-h thermal treatment at 150 °C allowed for the detection of seven major IL degradation products, presented in the Supplementary Material (Table S1). Though the main signals on the ^1^H-^13^C HSQC spectrum resonated from the IL cation and anion, the lower intensity signals, belonging to 1-methylimidazole, 1-butylimidazole, acetamides, and acetic acid esters, were reliably detected. This is an additional confirmation of the results obtained by GC-MS and IL’s degradation pathways presented in Figure 2. Among the detected compounds, there are three alkyl derivatives of imidazole, which are obviously the products of bmim interactions with the transfer of alkyl groups—1-butyl-2,3-dimethyl-1H-imidazol-3-ium cation, 1-butyl-2-methyl-1H-imidazole, and, surprisingly, 1-butyl-2-ethyl-1H-imidazole. This indicates the possibility of the formation in noticeable amounts of other low-volatile and thermolabile compounds, which cannot be detected by GC-MS, whereas NMR sensitivity may not be enough to identify them in complex mixtures.

To overcome this problem, an HPLC-HRMS non-target screening approach was applied to the analysis of ILs after the thermal treatment. The obtained chromatograms (Supplementary Figure S3) contain up to 25 peaks belonging to nitrogen-containing compounds with molecular weights of up to 305 Da (Supplementary Table S2). Accurate mass measurements have revealed the presence in their structures of 2 or 4 nitrogen atoms and up to 19 carbon and 2 oxygen atoms, which assumes the alkylation of the bmim cation, formation of dimers, and oxidation of the resulting compounds. As expected, the greatest variety of IL transformation products is characteristic of bmim acetate. In addition to compounds detected by GC-MS and NMR, the list of detected compounds includes three groups of homologues with high signal intensities that attract special attention. Depending on the elemental compositions and transformation degree, they were designated as A1-A2, B1-B3, and C1-C2 (Table 3).

**Figure 3:**
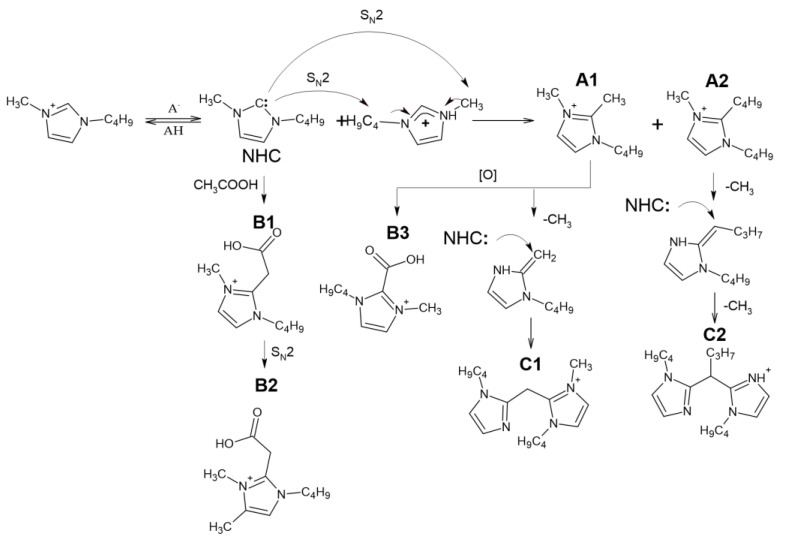
Assumed bmim degradation pathways leading to formation of less volatile products.

The obtained high-resolution accurate mass tandem (MS^2^ and MS^3^) mass spectra of these IL transformation products (Table S3) allowed for their tentative identification and assumptions about the reaction pathways (Figure 3). First of all, it should be noted that all the studied precursor ions are characterized by the easy loss of the butyl radical (elimination of butylene) during collision-induced dissociation (CID), indicating the presence of a butylimidazole fragment in their structure. In the case of A1, further fragmentation is achieved only in MS^3^, and leads to cleavage of the imidazole ring and elimination of the acetonitrile molecule. Compound A2 differs from A1 in the loss of one more butyl radical in the MS^3^ spectrum. Based on these data, A1 and A2 were identified as 1-Butyl-2,3-dimethyl-1H-imidazol-3-ium and 1,2-Dibutyl-3-methyl-1H-imidazole-3-ium cations, respectively.

The tandem mass spectra of oxygenated compounds, B1 and B3, are distinguished with the elimination of carbon dioxide from the debutylated precursor ion proving the presence of a carboxyl group in their structures. The fragmentation patterns indicate that in the case of B1, the COOH group is bound to the imidazolium via the -CH_2_- fragment (the loss of acetic acid), whereas in the structure of B3, it is attached directly to the cycle. Compound B2, which differs from B1 by an additional methyl group in its elemental composition, deserves special attention. Since its CID proceeds through elimination of butyl substituent and acetic acid (or CO_2_ in MS^3^ spectrum of debutylated ion), this compound cannot be the methyl ester of B1, and probably contains an additional methyl substituent in the imidazole ring. Our explanation of this phenomenon assumes that in the presence of substituents in the second position of the imidazole ring at elevated temperatures, deprotonation of the carbon atoms in the third and fourth positions with their subsequent alkylation by the S_N_2 mechanism is also possible. These reactions lead to the formation of a variety of alkyl derivatives observed in the obtained chromatograms.

The observed compounds have not been previously described in the literature, and the mechanism of their formation has not yet been fully elucidated. The presence of additional butyl and methyl radicals in A1 and A2 suggested their formation due to dealkylation of the bmim cation. Obviously, the formation of NHC initiates these interactions. The resulting intermediate NHC is quite reactive and has nucleophilic properties. Being a reactive nucleophile, NHC undergoes alkylation by the bmim cation via S_N_2 nucleophilic substitution to form A1 and A2. Similar reactions occur when the anion interacts with the bmim cation at temperatures above 120 °C [11]. Further oxidation of A1 leads to the formation of B3. Compound B1 is likely to be formed as a result of interaction with acetic acid or acetate anion with bmim carbene by analogy, with the reactions described earlier for pyridinium cycles [26]. However, this interaction resulted in the formation of a transient zwitter ion rather than a stable compound.

The presence of four nitrogen atoms in the elemental compositions of the products, C1 and C2, indicates the formation of dimeric bmim derivatives. Their tandem mass spectra contain the signals of product ions formed by the detachment of the butyl radical and 1-butylimidazole moiety. This allowed for the conclusion that C1 and C2 can be considered dimerization products of A1 and A2, respectively.

### 2.3. Thermal Stability of ILs

**Figure 4:**
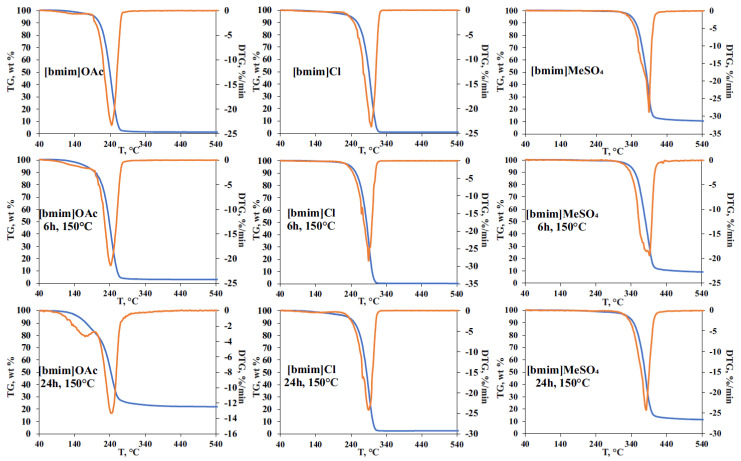
TG curves for initial and thermally treated at 150 °C for 6- and 24-h IL samples.

The effect of pre-heating on the thermal stability of ILs was studied by STA involving thermal gravimetric (TG), differential scanning calorimetry (DSC), and on-line electron ionization (EI) mass spectrometry of evolved gases. The obtained TG curves for the initial and treated IL samples (Figure 4), as expected, showed the formation of two weight loss ranges. The first one (140–220 °C) is common for all studied ILs, and is related mainly to evaporation of accumulated volatile degradation products. EI-MS measurements within this temperature range (Figure S4) allowed for the detection of ammonia, which can be the product of either deep bmim degradation or EI-induced fragmentation of amines. The observed weight loss at this stage was most pronounced for [bmim]OAc, and reached 5 and 20% for 6 h and 24 h treatments, respectively, which was in a good agreement with the vacuum drying results (Figure 1). Naturally, bmim chloride and, especially, methyl sulfate demonstrated much lower weight loss. However, differential scanning calorimetry (DSC) measurements revealed significant heat flows (endothermic peaks) within this temperature range (Figure S5a).

The second stage on TG curves (weight loss range) corresponds to the thermal decomposition of ILs and the formed less-volatile degradation products. In the case of [bmim]OAc, pre-heating at 150 °C did not cause a noticeable change in the decomposition temperature and the shape of the peak on the differential TG curve. Mass spectrometry detection of the evolved products in the selected fragment ion monitoring mode (Figure S4a) demonstrated the synchronous formation of methyl acetate (*m/z* 59), butyl acetate (*m/z* 73), and 1-butylimidazole and 1-methylimidazole (*m/z* 55 and 42). The fragment ions with *m/z* 41, 32, 31, and 30 indicate the formation of methylamine, butylamine, methanol, and butanol, proving the results obtained by Py-GC-MS. Special attention should be drawn to the effect of thermal treatment on the total weight loss during TG analysis. Though the initial (non-treated) [bmim]OAc sample is characterized with a complete transformation to volatile products in the temperature range of 200–300 °C (the maximum decomposition rate was observed at 242 °C), the residual weight of 3 and 22% was registered for the samples pre-heated for 6 and 24 h, respectively. The obtained residue was identified as a carbon which is likely to be formed with the participation of less volatile and more condensed bmim degradation products, as described in Section 2.2.

Being less prone to NHC formation, [bmim]Cl undergoes the most rapid decomposition at a higher temperature (~295 °C), and the carbon residue obtained in our experiments did not exceed 3% even for the sample pre-heated for 24 h. Nevertheless, the pre-heating effect on IL thermal stability caused by the accumulation of IL’s degradation products was observed—the temperature corresponding to the maximum decomposition rate (T_max_) was decreased from 298 to 290 °C after the thermal treatment at 150 °C. It is worth noting that, in contrast to [bmim]OAc, the presence of degradation products in [bmim]Cl leads to a more complex DTG peak shape having a reproducible shoulder at 294 °C. In addition to 1-butylimidazole and 1-methylimidazole, the intense signals of chloromethane (*m/z* 50) and 1-chlorobutane (fragment at *m/z* 56) were observed within the temperature range of 240–320 °C (Figure S4b). An interesting fact is that the process of pre-heated [bmim]Cl thermal decomposition occurs in three stages according to mass spectrometry and DSC measurements (Figures S4b and S5b), and involves, in addition to the evaporation/degradation of volatile compounds and the main stage of IL’s pyrolysis, the decomposition of small amounts of some condensed products (probably oligomers) at 390–440 °C, with the formation of 1-butylimidazole and 1-methylimidazole.

The phenomenon of multi-stage thermal decomposition is even more characteristic of [bmim]MeSO_4_ having the anion of low volatile acid. As in the case of bmim chloride, T_max_ of this IL decreases by 8 °C (from 390 to 382 °C) after 24-h pre-heating at 150 °C. The main DTG peak covering the temperature range of 320–440 °C consists of the three zones clearly visible in DSC and selected ion monitoring graphs (Figures S4c and S5c). The first one is located within the range of 320–370 °C and contains the major signals of 1-methylimidazole (*m/z* 42), 1-methoxybutane (*m/z* 45), and 1-butanol (*m/z* 43) fragment ions. The second zone (380–410 °C) involves an active decomposition of IL’s anion, and is characterized by the formation of sulfur dioxide (*m/z* 64) and 1-butylimidazole (fragment ion at *m/z* 55). The third zone is located at 410–440 °C, and is distinguished with the formation of water (*m/z* 18) and methylamine (*m/z* 31) as final products of the decomposition of the most stable compounds, most likely occurring at high temperatures due to the action of admixtures accumulated during IL pre-heating. The higher temperatures of [bmim]MeSO_4_ decomposition and the presence of a low-volatile anion result in the formation of carbon residue in amounts of 10 and 14% for the initial and pre-heated (150 °C, 24 h) samples, respectively.

## 3. Materials and Methods

### 3.1. Reagents and Materials

The ionic liquids, 1-butyl-3-methylimidazolium acetate, methyl sulfate, and chloride (BASF quality, >95%), were purchased from Sigma Aldrich (Schnelldorf, Germany). Deionized Milli-Q water and HPLC gradient-grade acetonitrile (Cryochrom, S.-Petersburg, Russia) were used for sample preparation and as components of the mobile phase in HPLC-MS experiments.

### 3.2. Thermal Treatment and Sample Preparation

Two samples (1.0 g) of each IL were placed in 20 mL air-tight vials with steel screwcaps and PDMS/PTFE septa, and were incubated in an oven at 150 °C for 6 or 24 h, with further cooling at room temperature without opening. The samples of initial ILs were prepared in the same manner without thermal treatment. One sample of each IL thermally treated for the specified time was transferred to the GC-MS system autosampler without vial decapping, and was subjected to HS-GC-MS analysis. Four accurately weighed portions were carefully taken from another IL sample. The first one (0.5 mg) was placed in a pyrolysis glass liner insert (30 µL micro vial) for further Py-GC-MS analysis. Another one was dissolved in an acetonitrile–water (1:1) mixture, and diluted with the same solvent to obtain a solution with a concentration of 100 mg L^−1^, which was further subjected to HPLC-HRMS analysis. One more portion (20–30 mg) of IL was transferred to an alumina micro crucible for further STA analysis. The fourth portion (100 mg) was dissolved in 0.5 mL of DMSO-d_6_ and used for NMR analysis.

### 3.3. GC-MS Analyses

A GC-MS system, consisting of a GCMS-TQ8040 gas chromatograph with a triple quadrupole tandem mass spectrometer (Shimadzu, Kyoto, Japan), Optic4 temperature programmable (PTV) injector with a liquid nitrogen cooling cryo-trap (GL Sciences, Eindhoven, Netherlands), and an AOC-5000 Plus robotic autosampler (Shimadzu, Kyoto, Japan), was used for headspace and pyrolysis GC-MS analyses. Instrument control, data collection, and primary processing were performed using LabSolutions software (Shimadzu, Kyoto, Japan). The temperature of the PTV injector was programmed using Optic4 Evolution software (GL Sciences, Eindhoven, Netherlands). Chromatographic separation was achieved on an HP-INNOWax (Agilent Technologies, Santa Clara, CA, USA) fused silica capillary column (30 m, 0.25 mm i.d. and 0.25 µm film thickness) with a polyethylene glycol stationary phase. High-purity (99.9999%) helium (NIIKM, Moscow, Russia) was used as a carrier gas, with a flow rate of 1.2 mL min^−1^. The oven temperature was programmed from 40 °C (held for 3 min) to 240 °C (held for 5 min), with a 10 °C min^−1^ ramp. The total duration of the analysis was 28 min. Mass spectrometry detection was conducted using electron ionization (70 eV) in scan mode (*m/z* 35–350). The transfer line temperature was 240 °C, the ion source temperature was 230 °C, and the detector voltage was 1.07 kV. The identification of analytes was performed using an NIST-14 mass spectral library (match factor > 800).

Difficult matrix introduction (DMI) tapered liner (GL Sciences, Eindhoven, Netherlands), with indentations to hold the 30 µL micro vial with the IL sample, was used in Py-GC-MS experiments. The following PTV injector temperature program was used: 1 min held at 35 °C, 60 °C s^−1^ ramp to specified pyrolysis temperature, held for 10 min. The injection of pyrolysis products was carried out in split (100:1) mode. A temperature-programmable cryo-trap, connected directly to the chromatographic column, was used for cryo-focusing the pyrolysis products at −100 °C throughout the entire thermal desorption procedure, and introducing them into GC column by rapid heating to 200 °C.

Headspace GC-MS analysis of thermally treated IL samples was carried out in 20 mL headspace vials containing 1.0 g of IL using robotic autosampler. After 1-h equilibration at 100 °C in an agitator with continuous shaking, 0.5 mL of the vapor phase was taken with a gas-tight syringe heated to 100 °C, and then injected in splitless mode into the GC column. No cryo-focusing was used.

### 3.4. NMR Spectroscopy

NMR spectra were registered on an AVANCE III NMR spectrometer (Bruker, Ettlingen, Germany) with an operational frequency of 600 MHz for protons. The experiment parameters to register the 2D (^1^H-^13^C) HSQC (Heteronuclear Single Quantum Correlation) spectrum were: temperature—298 K, spectrum window width ~13 ppm for F2, and ~200 ppm for F1, with number of accumulations 1024 × 256, number of scans—8. The delay time between pulses (D1) was 2.0 s. The experiment parameters for HMBC were: temperature—298 K, spectrum window width ~12 ppm for F2, and ~240 ppm for F1, with number of accumulations 2048 × 512, number of scans—8. The delay time between pulses (D1) was 2.0 s. The assignment of cross-peaks and tentative identification of analytes were carried out using an ACD/Labs computational expert system (ACD/Labs, Toronto, ON, Canada).

### 3.5. HPLC-HRMS Analysis

The HPLC-HRMS study was performed using a high-resolution “tribrid” mass spectrometry system, Orbitrap ID-X (Thermo Scientific, Waltham, MA, USA), with OptaMax NG ion source combined with an LC-30 Nexera HPLC system (Shimadzu, Kyoto, Japan) consisting of two LC-30AD HPLC pumps, vacuum degasser, an STO-30A column thermostat, and an SIL-30AC autosampler. Chromatographic separation was carried out on a Nucleodur PFP column, 150 × 3 mm, 1.8 μm particle size (Macherey-Nagel, Duren, Germany), with the pentafluorophenyl stationary phase in gradient elution mode. Acetonitrile (A) and water (B) were used as mobile phase components. The following gradient program was used: 0–2 min—10% A, 2–15 min—linear ramp to 100% A, 15–20 min—100% A; injection volume—2 µL, mobile phase flow rate—0.25 mL min^−1^, column temperature—40 °C.

Mass spectrometry detection was carried out using electrospray ionization in positive ion mode (ESI+). The ion source parameters were as follows: spray voltage—3.5 kV; sheath, auxiliary and sweep gas (N_2_) flow rates—50, 10, and 2 arb. units, respectively; ion transfer tube temperature, 325 °C; vaporizer temperature, 350 °C; S-lens RF level, 60 arb. units. Data dependent acquisition mode was used in the non-target analysis. The signal intensity threshold of 1.0 × 10^5^ cps was used as a criterion for quadrupole isolation and higher-energy collision-induced dissociation (HCD) of precursor ions. Tandem (MS/MS) mass spectra were obtained using a collision energy of 20 eV (collision gas—nitrogen). A mass analyzer resolving power of 120,000 and 30,000 was used in MS and MS/MS modes, respectively. The scanning range was *m/z* 100–1000 in MS mode and was set to “auto” in MS/MS mode. Xcalibur software (Thermo Scientific, Waltham, MA, USA) was used to control the instrument and acquire HRMS chromatograms. Non-target screening and the identification of detected compounds were carried out using the Compound Discovery software package (Thermo Scientific, Waltham, MA, USA), providing an online search in the Chemspider and *m/z* Cloud databases. In elemental composition calculations, the maximum allowed deviation of the measured *m/z* from the theoretical value was 5 ppm.

### 3.6. Synchronous Thermal Analysis

STA analysis was performed using an STA 449 F3 Jupiter thermal analysis system (Netzsch, Selb, Germany) combined with an Aeolos QMS 403 CF (Netzsch, Selb, Germany) quadrupole mass spectrometer with an electron ionization (70 eV) ion source, through a heated (250 °C) transfer line quartz capillary. Before starting measurements, the microbalance was calibrated with the built-in calibration weight, and the system was calibrated for temperature and enthalpy. For this purpose, standard calibration samples of high-purity metals were used: indium, tin, bismuth, zinc, aluminum, and gold. All experiments were performed in dynamic mode: the sample chamber was continuously purged with high-purity (99.995%) argon at a flowrate of 40 mL min^−1^ during measurements. The baseline was measured immediately before experiments using empty crucibles. The measurements were carried out in the heating mode from 40 to 900 °C at a rate of 10 °C min^−1^, and an empty crucible of the same material was used as a reference sample. Mass spectra of the gaseous thermal decomposition products were recorded continuously throughout the experiment. Proteus software (Netzsch, Selb, Germany) was used for instrument control and the acquisition of TG, DSC, and mass spectrometry data.

## 4. Conclusions

Thermal treatment of 1-butyl-3-methylimidazolium-based ILs at 150 °C, a typical temperature for lignocellulosic biomass dissolution, leads to a gradual decomposition of ILs and the accumulation of a variety of volatile and non-volatile thermal degradation products in amounts ranging from 3 to 25% by weight after 24-h heating. Among them, a number of alkyl-substituted imidazoles, including dimeric compounds, methanol and butanol, alkyl amines, methyl and butyl acetates and N-alkylamides (in the case of [bmim]OAc]), and chloroalkanes (in the case of [bmim]Cl]) predominate. Many IL degradation products were identified for the first time in the present study. The key role in ILs’ thermal behavior belongs to the proton transfer from bmim cation to anion with the formation of reactive N-heterocyclic carbene (NHC), which undergoes further reactions with anion or imidazole ring cleavage. By thermal lability, ILs can be arranged in the following sequence, coinciding with the decrease in basicity of the anion: [bmim]OAc > [bmim]Cl > [bmim]MeSO_4._ The accumulation of thermal degradation products in ILs, in turn, affects their physico-chemical properties and thermal stability, and leads to a slight decrease in the decomposition temperature, a change in the shape of the TG curves, and the formation of carbon residue during pyrolysis. These features must be considered when developing IL technologies for processing plant biomass, and in the regeneration and reuse of ILs.

## Figures and Tables

**Table 1:** Results of HS-GC-MS analysis of volatile and semi-volatile degradation products in IL samples before (0-h) and after 6- and 24-h thermal treatment at 150 °C.

Compound	Chromatographic Peak Area, arb. Units ×10^2^								
	[bmim]OAc			[bmim]Cl			[bmim]MeSO_4_		
	0 h	6 h	24 h	0 h	6 h	24 h	0 h	6 h	24 h
1-Metanol	950	3200	18,000	– *	110	1200	–	16,900	19,000
1-Butanol	–	43	40	–	–	–	–	–	–
Acetic acid methyl ester	1	13,700	18,000	–	–	–	–	–	–
Acetic acid butyl ester	–	86	77	–	–	–	–	–	–
1-Chloromethane	–	–	–	–	2400	4300	–	–	–
1-Chlorobutane	–	–	–	–	87	190	–	–	–
1-Butylimidazole	–	75	470	–	2	7	–	–	–
1-Methyl-1H-Imidazole	6	250	2900	–	29	55	1	2	2
N-methyl-Formamide	–	15	1100	–	–	–	–	–	–
N-methyl-Acetamide	–	1	11	–	–	–	–	–	–
Trimethylamine	–	160	187	–	–	–	–	–	–
N,N-Dimethylbutylamine	–	24	41	–	–	–	–	–	–

*—not detected.

**Table 2:** Products of bmim acetate, chloride, and methyl sulfate pyrolysis at 242, 290, and 363 °C, respectively, identified by Py-GC-MS before (0-h) and after 6- and 24-h thermal treatment at 150 °C.

Compound	Chromatographic Peak Area, arb. Units, ×10^3^								
	[bmim]OAc			[bmim]Cl			[bmim]MeSO_4_		
	0 h	6 h	24 h	0 h	6 h	24 h	0 h	6 h	24 h
Acetic acid methyl ester	100	110	140	– *	–	–	–	–	–
Acetic acid butyl ester	31	63	92	–	–	–	–	–	–
1-Methyl-1H-Imidazole	24	35	72	270	350	390	2200	2900	3000
1-Butylimidazole	640	1200	1600	2300	2900	2900	2800	3400	4300
N-methyl-Acetamide	–	1	8	–	–	–	–	–	–
N-butyl-Acetamide	–	5	23	–	–	–	–	–	–
N-butyl-Formamide	–	6	15	–	–	–	–	–	–
1-Butanol	3	5	7	–	1	1	130	130	140
Methanol	–	1	2	–	1	1	150	160	260
1-Chlorobutane	–	–	–	220	280	280	–	–	–
Chloromethane	–	–	–	5	6	6	–	–	–
1-Methoxybutane	–	–	–	–	–	–	–	5	5
Sulfur dioxide	–	–	–	–	–	–	2	82	84

*—not detected.

**Table 3:** The main low-volatile IL degradation products identified by HPLC-HRMS before (0-h) and after 6- and 24-h thermal treatment at 150 °C.

Compound	Formula	*m/z*	RT, min	Chromatographic Peak Area, arb. Units, ×10^7^		
				0 h	6 h	24 h
[bmim]OAc						
A1	[C_9_H_17_N_2_]^+^	153.1386	2.56	39	57	210
A2	[C_12_H_23_N_2_]^+^	195.1854	7.54	7	1700	2900
B1	[C_10_H_17_N_2_O_2_]^+^	197.1284	3.18	8	23	38
B2	[C_11_H_19_N_2_O_2_]^+^	211.1803	6.68	260	1100	2300
B3	[C_9_H_15_N_2_O_2_]^+^	183.1128	2.61	8	4300	5000
C1	[C_16_H_27_N_4_]^+^	303.2543	9.79	6	250	560
C2	[C_18_H_31_N_4_]^+^	275.2232	8.93	0	5	97
1-Butylimidazole	[C_7_H_13_N_2_]^+^	125.1073	1.79	110	5100	13,000
1-Butyl-2-Methylimidazole	[C_8_H_15_N_2_]^+^	139.1228	2.12	5	180	140
Butyl acetate	[C_6_H_13_O_2_]^+^	117.1005	5.57	22	310	1200
[bmim]Cl						
A2	[C_12_H_23_N_2_]^+^	195.1856	7.92	0	1	14
1-Butylimidazole	[C_7_H_13_N_2_]^+^	125.1073	1.79	56	860	1200
[bmim]MeSO_4_						
1-Butyl-2-Methylimidazole	[C_8_H_15_N_2_]^+^	139.1228	2.12	2	13	38

## Data Availability

Not applicable.

## Supplementary Materials

The following supporting information can be downloaded at: https://www.mdpi.com/article/10.3390/ijms231810966/s1.
