# Supplementary material for: New Insights into the Thermal Stability of 1-Butyl-3-methylimidazolium-Based Ionic Liquids
Source: Int J Mol Sci. 2022 Sep 19;23(18):10966. doi: 10.3390/ijms231810966 (PMC9502186; doi:10.3390/ijms231810966)
Supplement: Supplementary file 1 [file ijms-23-10966-s001.zip › ijms-1932049-supplementary.pdf]

Supplementary Materials

New Insights into the Thermal Stability of 1-Butyl-3-methylimidazolium-based Ionic Liquids

Artyom V. Belesov, Natalya V. Shkaeva, Mark S. Popov, Tatyana E. Skrebets, Anna V. Faleva, Nikolay V. Ul'yanovskii and Dmitry S. Kosyakov*

Core Facility Center "Arktika", Northern (Arctic) Federal University, nab. Severnoy Dviny, 17, Arkhangelsk, 163002, Russia
* Correspondence: d.kosyakov@narfu.ru

Contents:

Figure S1. Aliphatic region of ^{1}H-^{13}C HSQC spectrum of [bmim]OAc.

Figure S2. Aromatic region of ^{1}H-^{13}C HSQC spectrum of [bmim]OAc.

Figure S3. Extracted ion chromatograms for [bmim]OAc (24 h, 150°C).

Figure S4. Selected ion mass spectrometry evolved gas analysis in STA experiments (a-[bmim]OAc; b-[bmim]Cl; c-[bmim]$MeSO_4$).

Figure S5. Differential scanning calorimetry measurements (a-[bmim]OAc; b-[bmim]Cl; c-[bmim]$MeSO_4$).

Table S1. Components identified on ^{1}H-^{13}C HSQC spectrum of [bmim]OAc heated to 150°C.
Table S2. The low-volatile IL degradation products identified by HPLC-HRMS before (0-h) and after 6- and 24-h thermal treatment at 150°C.

Table S3. Mass spectra of IL major degradation products detected by HPLC-HRMS.

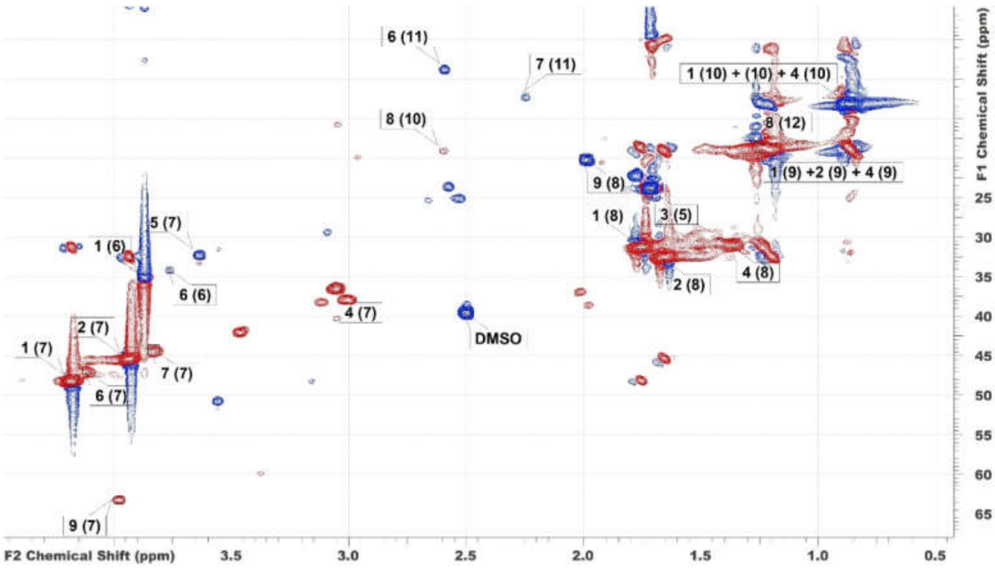

Figure S1. Aliphatic region of ^{1}H-^{13}C HSQC spectrum of [bmim]OAc.

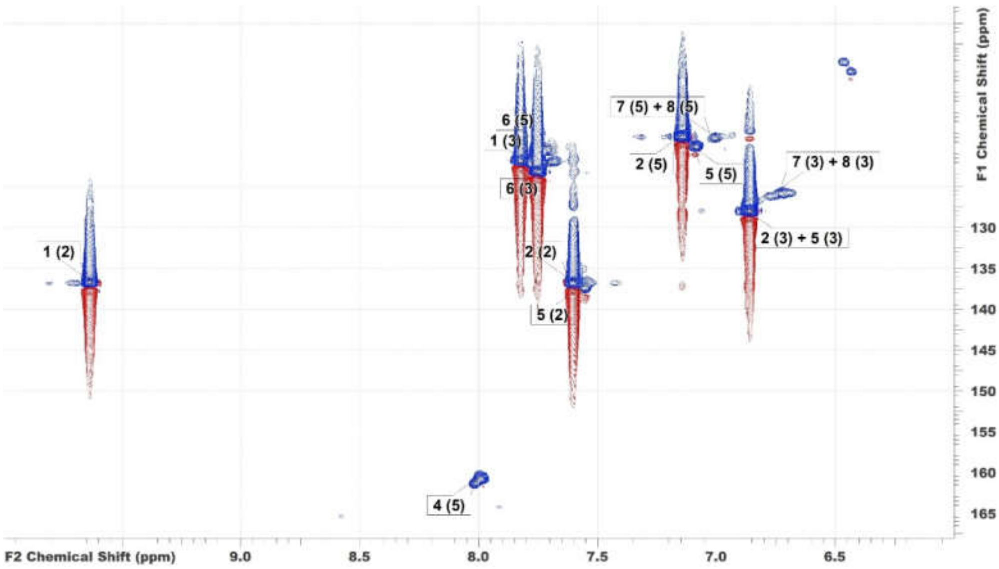

Figure S2. Aromatic region of ^{1}H-^{13}C HSQC spectrum of [bmim]OAc.

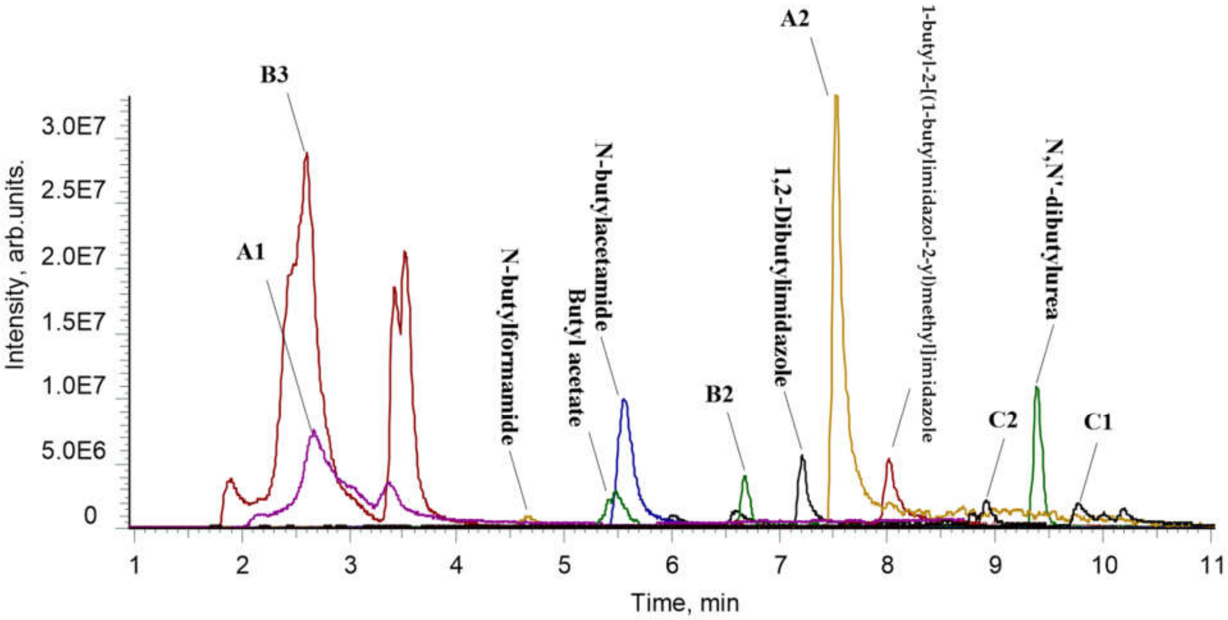

Figure S3. Extracted ion chromatograms for [bmim]OAc (24 h, 150°C).

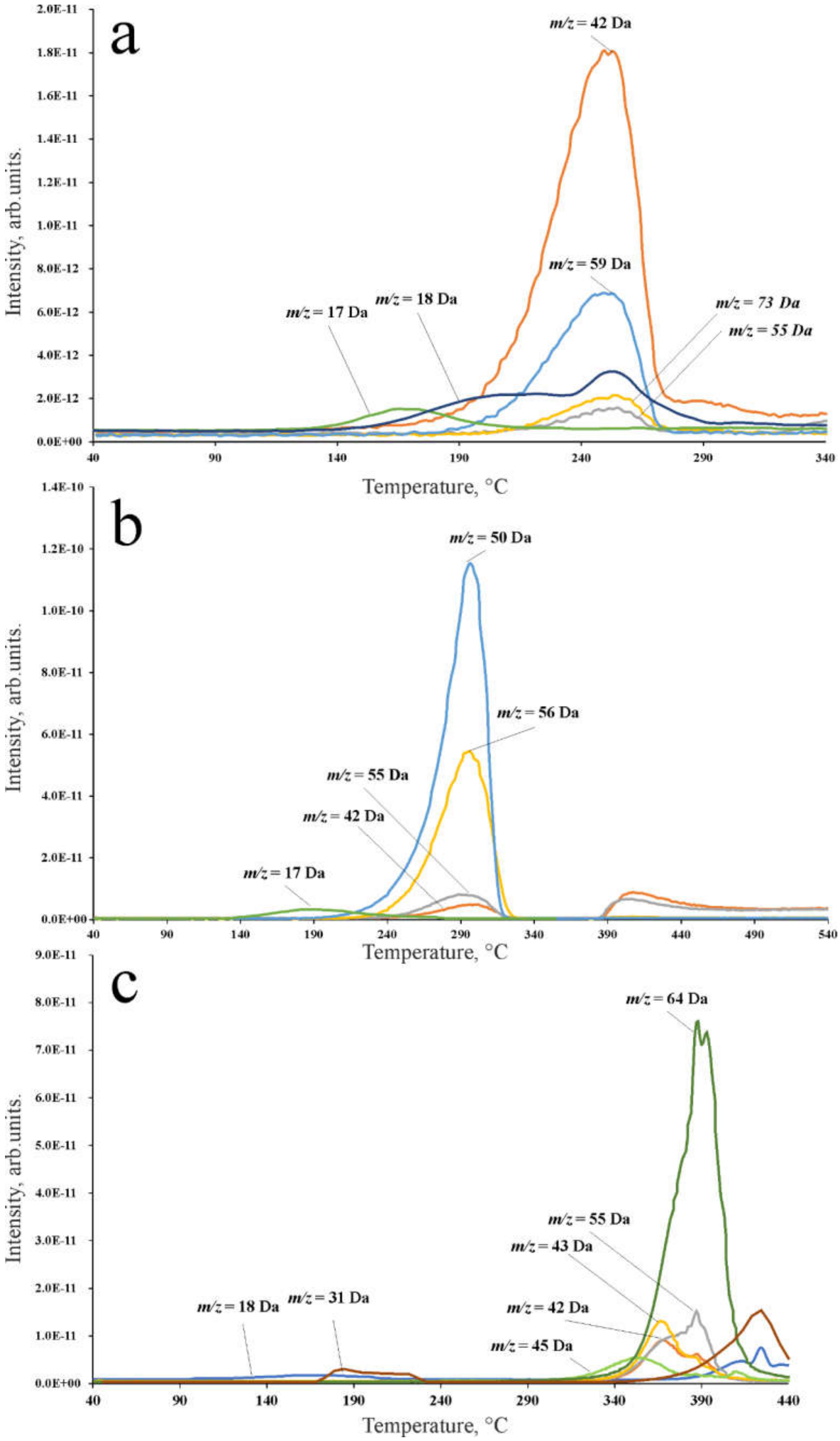

Figure S4. Selected ion mass spectrometry evolved gas analysis in STA experiments (a-[bmim]OAc; b-[bmim]Cl; c-[bmim]MeSO4).

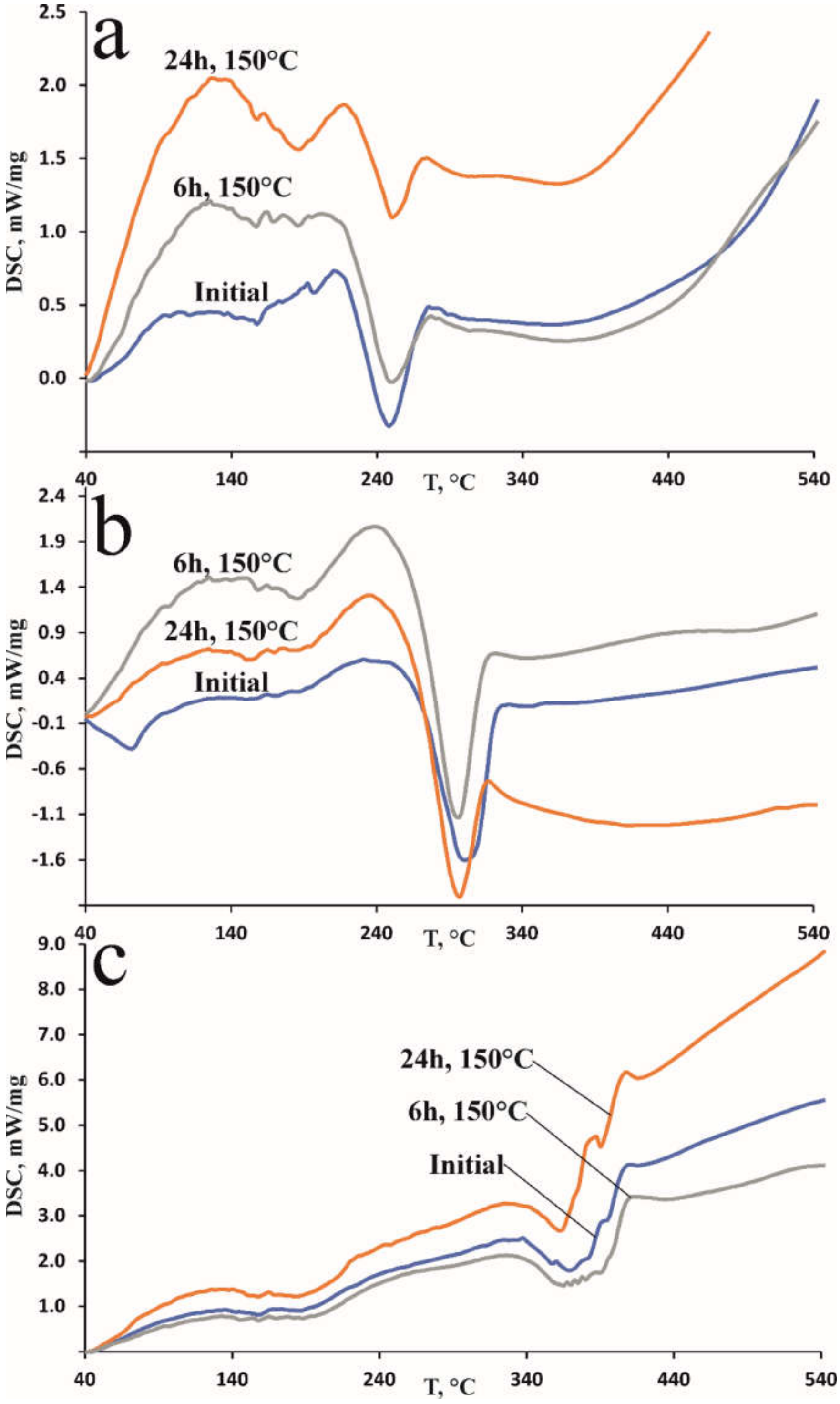

Figure S5. Differential scanning calorimetry measurements (a-[bmim]OAc; b-[bmim]Cl; c-[bmim]$MeSO_4$).

Table S1. Components identified on ^{1}H-^{13}C HSQC spectrum of [bmim]OAc heated to 150°C.

#	Structure	Formula	FW
1	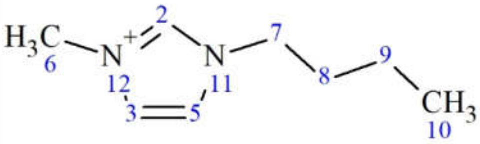	$C_8H_{15}N_2^+$	139.2176
2	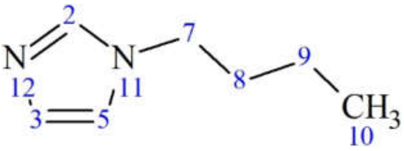	$C_7H_{12}N_2$	124.1836
3	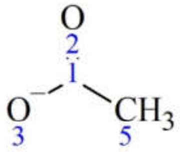	$C_2H_3O_2^-$	59.0446
4	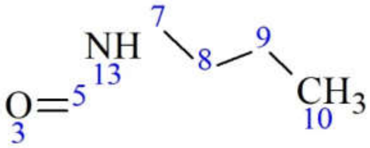	$C_5H_{11}NO$	101.1469
5	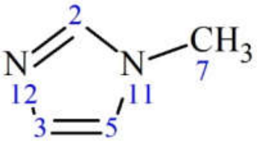	$C_4H_6N_2$	82.1038
6	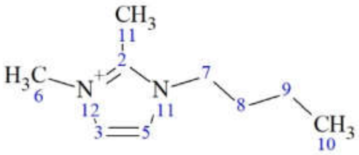	$C_9H_{17}N_2^+$	153.2441
7	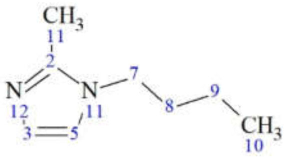	$C_8H_{14}N_2$	138.2102
8	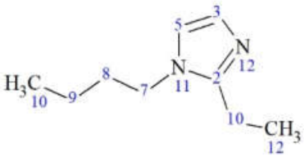	$C_9H_{16}N_2$	152.2367
9	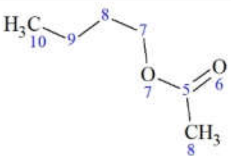	$C_6H_{12}O_2$	116.1583

Table S2. The low-volatile IL degradation products identified by HPLC-HRMS before (0-h) and after 6- and 24-h thermal treatment at 150°C.

Compound	Formula	*m/z*	*Δ, ppm*	RT, min	Chromatographic peak area, arb. Units ·10^6		
					0 h	6 h, 150°C	24 h, 150°C
[bmim]OAc							
A1	$[C_9H_{17}N_2]^+$	153.1386	0.29	2.56	4	6	21
A2	$[C_{12}H_{23}N_2]^+$	195.1854	0.97	7.54	1	172	288
B1	$[C_{10}H_{17}N_2O_2]^+$	197.1284	0.36	3.18	1	2	4
B2	$[C_{11}H_{19}N_2O_2]^+$	211.1803	0.30	6.68	26	106	233
B3	$[C_9H_{15}N_2O_2]^+$	183.1128	0.92	2.61	1	429	499
C1	$[C_{16}H_{27}N_4]^+$	303.2543	0.14	9.79	1	25	56
C2	$[C_{18}H_{31}N_4]^+$	275.2232	0.88	8.93	0	1	10
1-Butylimidazole	$[C_7H_{13}N_2]^+$	125.1073	0.51	1.79	12	510	1344
1-Butyl-2-methylimidazole	$[C_8H_{15}N_2]^+$	139.1228	0.75	2.12	1	19	14
Butyl acetate	$[C_6H_{13}O_2]^+$	117.1005	1.68	5.57	2	31	117
N-butylformamide	$[C_5H_{12}NO]^+$	102.0913	0.08	4.66	0	12	14
N-butylacetamide	$[C_6H_{14}NO]^+$	116.1069	0.78	5.58	3	34	125
2,4,5-Trimethyl-1-butylimidazole	$[C_{10}H_{19}N_2]^+$	167.1542	0.63	3.75	0	189	340
N,N'-dibutylurea	$[C_9H_{21}N_2O]^+$	173.1647	0.72	9.41	9	106	178
1-butyl-2-[(1-butylimidazol-2-yl)methyl]imidazole	$[C_{15}H_{25}N_4]^+$	261.2074	0.09	8.04	0	51	66
1,2-Dibutylimidazole	$[C_{11}H_{21}N_2]^+$	181.1699	0.26	7.24	0	8	44
[bmim]Cl							
A1	$[C_9H_{17}N_2]^+$	153.1386	0.60	3.07	0	12	14
A2	$[C_{12}H_{23}N_2]^+$	195.1856	0.17	7.92	0	0	1
1-Butylimidazole	$[C_7H_{13}N_2]^+$	125.1073	0.87	1.79	6	86	123
1,2-Dibutylimidazole	$[C_{11}H_{21}N_2]^+$	181.1698	0.89	7.30	2	12	194
[bmim]MeSO$_4$							
1-Butyl-2-methylimidazole	$[C_8H_{15}N_2]^+$	139.1228	0.75	2.12	0	1	4

Table S3. Mass spectra of IL major degradation products detected by HPLC-HRMS.

Compound	RDB	m/z, Da	MS^2	MS^3
A1	3	153.1385	153.1384($C_9H_{17}N_2$) - 100%<br>*97.0758($C_5H_9N_2$) - 79%	97.0758($C_5H_9N_2$) - 100%<br>53.0494(C_3H_6N) - 22%
A2		195.1854	195.1854($C_{12}H_{23}N_2$) - 100%<br>*138.0660($C_8H_{14}N_2$) - 15%<br>139.1228($C_8H_{15}N_2$) - 5%	138.0660($C_8H_{14}N_2$) - 100%<br>83.0602($C_4H_7N_2$) – 5%
B1	4	197.1284	197.1284($C_{10}H_{17}O_2N_2$) - 100%<br>*141.0657($C_6H_9O_2N_2$) - 30%<br>137.1072($C_8H_{13}N_2$) - 5%<br>123.0914($C_7H_{11}N_2$) - 4%	141.0657($C_6H_9O_2N_2$) - 100%<br>97.0758($C_5H_9N_2$) - 31%<br>56.0494(C_3H_6N) - 18%
B2		211.1441	211.1441($C_{11}H_{19}O_2N_2$) - 100%<br>*155.0814($C_7H_{11}O_2N_2$) - 22%<br>151.1229($C_9H_{15}N_2$) - 4%<br>137.1072($C_8H_{13}N_2$) - 5%	155.0814($C_7H_{11}O_2N_2$) – 100%<br>137.0708($C_7H_9ON_2$) – 12%<br>111.0914($C_6H_{11}N_2$) – 23%<br>83.0602($C_4H_7N_2$) – 5%
B3		183.1127	183.1126($C_9H_{15}O_2N_2$) - 100%<br>*127.0500($C_5H_7O_2N_2$) - 50%	127.0500($C_5H_7O_2N_2$) - 100%<br>109.0395($C_5H_5ON_2$) - 21%<br>95.0238($C_4H_3ON_2$) - 4%<br>83.0602($C_4H_7N_2$) - 48%
C1	7	303.2544	303.2544($C_{18}H_{31}N_4$) – 100%<br>*247.1917($C_{14}H_{23}N_4$) – 8%<br>179.1542($C_{11}H_{19}N_2$) – 17%<br>137.1074($C_8H_{13}N_2$) – 4%	247.1917($C_{14}H_{23}N_4$) – 100%<br>191.1290($C_{10}H_{15}N_4$) – 60%
C2		275.2231	275.2231($C_{16}H_{27}N_4$) – 100%<br>*219.1604($C_{12}H_{19}N_4$) – 7%<br>163.0978($C_8H_{11}N_4$) – 2%<br>137.1072($C_8H_{13}N_2$) – 1%	219.1604($C_{12}H_{19}N_4$) – 100%<br>163.0976($C_8H_{11}N_4$) – 88%
